# Efficacy and Safety of Combination Therapy of Microneedling Radiofrequency, In‐Office and Home‐Based Topical Cysteamine in Refractory Melasma: A Split Face, Vehicle‐Control, Randomized Control Trial

**DOI:** 10.1111/jocd.16661

**Published:** 2024-11-22

**Authors:** Ya‐Wen Tsai, Jeng‐Hsien Lin, Yi‐Jing Lai, Tzu‐Li Liu, Chau Yee Ng

**Affiliations:** ^1^ School of Medicine, College of Medicine Chang Gung University Taoyuan Taiwan; ^2^ Department of Dermatology Chang Gung Memorial Hospital Taipei Linkou Taiwan; ^3^ Department of Dermatology and Aesthetic Medicine Center Jen‐Ai Hospital Tai Chung Taiwan; ^4^ Dr. LIN's Dermatological Clinic Kaohsiung City Taiwan; ^5^ Department of Internal Medicine Taipei Medical University Hospital Taipei Taiwan; ^6^ Vitiligo Clinic and Pigment Research Center Chang Gung Memorial Hospital Linkou Taiwan

**Keywords:** cysteamine, isobionic‐amide complex, melasma, microneedling radiofrequency, split‐face trial

## Abstract

**Background:**

Refractory melasma remains a challenge in dermatology, necessitating the exploration of innovative treatments.

**Aims:**

This study aims to evaluate the efficacy and safety of combining radiofrequency microneedling (RFM) with Cysteamine cream, applied both in‐office and as a part of a home‐care regimen, to treat refractory melasma.

**Patients/Methods:**

A vehicle‐controlled, split‐face trial was conducted on 30 Fitzpatrick skin types III and IV participants. Subjects received RFM and Cysteamine serum on one side of the face and RFM with saline on the contralateral side. Additionally, a random half‐added Cysteamine cream was applied at home. The modified Melasma Area and Severity Index and VISIA skin analysis were used for assessment.

**Results:**

Significant improvement in melasma severity and skin texture was noted on the sides treated with RFM and Cysteamine, including observable benefits from both in‐office and home‐based Cysteamine use with minimal adverse effects.

**Conclusions:**

The combined approach of RFM with Cysteamine provides a promising and safe modality for managing refractory melasma, showing notable improvements.

## 1 Introduction

Melasma is a common acquired hyperpigmentation, manifesting as symmetric, blotchy hypermelanosis, mainly observed in those with Fitzpatrick skin types III and IV. It is more commonly diagnosed in darker‐skinned women, particularly of Asian or Hispanic origin. The condition arises from the complex interaction among skin cells, including melanocytes, keratinocytes, and dermal fibroblasts, influenced by hormones, genetics, and UV radiation [1, 2]. The principal pathophysiological mechanisms implicated in melasma have been delineated as follows: first, the undue activation of melanocytes; second, the congregation of melanin and melanosomes within both the dermis and epidermis; third, a dual aspect involving an augmented count of mast cells and the occurrence of solar elastosis; fourth, modifications in the integrity of the basement membrane; and fifth, an enhancement in vascularization [3].

Melasma management is challenging due to its recurrent nature, with no universally effective single treatment for all cases. Treatment strategies often combine multiple approaches and aim to minimize factors that worsen the condition. Established treatments encompass topical applications like hydroquinone, Kojic acid, tranexamic acid, chemical peels, and laser treatments [2, 4, 5, 6, 7]. New methods, such as microneedling, have been introduced, targeting the multifaceted pathophysiology of melasma and providing new hope for more effective treatments through laser and light therapies.

Recently, microneedling has gained popularity due to its proven efficacy for various skin issues, including melasma, scars, and wrinkles [8]. The integration of various topical agents, notably topical tranexamic acid, has been proven to augment the efficacy of treatments for melasma [9]. Radiofrequency microneedling (RFM) refines the microneedling technique by incorporating radiofrequency energy. This innovation stimulates collagen production within the dermis, augments the absorption of skincare products, and promotes the release of growth factors crucial for skin rejuvenation. Initial research investigating the synergistic application of RFM alongside tranexamic acid and a triple combination cream for treating melasma has yielded encouraging outcomes [10]. On the contrary, a single trial in combination with polynucleotides has yielded negative results [11]. Further research is needed to confirm these initial findings and validate the effectiveness of the combined treatment approach.

Cysteamine, a natural compound in human breast milk with potent antioxidant properties, has shown promise in reducing melanin synthesis in the skin by inhibiting tyrosinase activity [6]. This makes it an effective treatment for melasma, akin to hydroquinone, but without the associated risk of ochronosis from long‐term use. Clinical trials have confirmed Cysteamine's ability to significantly diminish melasma within 8–16 weeks of consistent application [2, 12, 13, 14, 15, 16].

Combining RFM with cysteamine provides a synergistic effect in treating refractory melasma. The micro‐injuries created by RFM enhance the penetration of cysteamine into the dermis, allowing it to act more effectively by inhibiting tyrosinase, a key enzyme involved in melanin synthesis [9, 12, 17]. This combined approach not only addresses the overproduction of melanin but also promotes dermal remodeling and neocollagenesis, which improves skin texture and reduces pigmentation more effectively than either treatment alone. This randomized controlled split‐face study assessed and compared the efficacy of RFM alone versus RFM in combination with cysteamine.

## 2 Materials and Methods

### 2.1 Study Design

This randomized, split‐face controlled trial was conducted to evaluate the therapeutic efficacy and safety of a novel combined treatment regimen involving the use of a non‐insulated pulsed‐type bipolar RFM (Sylfirm, Viol Co. Ltd., Korea) and topical in‐office cysteamine serum for individuals with treatment‐resistant melasma. The RFM, as mentioned above, consists of a 10 × 10 mm disposable tip with 25 non‐insulated, penetrating microneedles in a uniform 5 × 5 array with surgical stainless steel, each 0.3 mm thick and 3.5 mm long. The whole face was treated with RFM energy settings at level II 1.5 mm penetration depth, delivered in uniform passes across the treated area, and repeated for two passes with a frequency of 2 MHz; each pass was done with 25% overlap. The subject's right side face received microneedling RF coupled with in‐office Cysteamine serum (Cyspera, Scientis Pharma, Switzerland), and the left side received RFM with in‐office placebo control (normal saline). Subjects were further stratified into two groups; one group received home‐based topical cysteamine cream (intensified Cyspera, Scientis Pharma, Switzerland) treatment as daily skin care, whereas the other group received a placebo‐vehicle control. The participants were instructed to use the home‐based topical skin care daily. The product was to be applied in the evening, left on the skin for 15 min, and then washed off. This regimen was continued until Day 180 as part of the home care routine. The microneedling treatment was delivered over four sessions at a monthly interval, followed by evaluations during the fifth and sixth visits.

The baseline treatment was established as Day 0, with follow‐up assessments on Days 30, 60, 90, 120, and 180.

### 2.2 Participants

Thirty participants with a clinical diagnosis of melasma were recruited at Linkou Chang Gung Memorial Hospital between December 1, 2022, and November 30, 2023. Inclusion criteria were a Fitzpatrick skin type III or IV, age over 20, and a history of refractory melasma. The exclusion criteria for participation in the study were carefully defined to include individuals who were pregnant, under the age of 20, under oral contraceptive or hormonal therapy, had used hydroquinone or tranexamic acid within the previous 3 months, or had received laser treatment within the same time frame. The study noted a dropout rate involving four participants, attributed to the inconvenience associated with the requirement for monthly visits.

### 2.3 Outcome Measures

The primary endpoint of the study was treatment efficacy, determined by the modified Melasma Area and Severity Index (m‐MASI), as evaluated by two independent dermatologists (Y.‐W.T. and C.Y.N.), and analysis of skin changes using the VISIA system, which measured wrinkles, texture, pore sizes, and pigment spots. The secondary endpoint was the assessment of potential rebound effects following treatment. A patient self‐reported improvement questionnaire was used and was scored during each visit. Documented adverse reactions, recorded at each visit, included erythema, scaling, itch, and pain.

### 2.4 Statistical Analysis

Data analysis was performed using SPSS software, version 29 (IBM, USA). The Wilcoxon signed‐rank test was employed to determine within‐subject improvements on each facial side before and after treatment. The Mann–Whitney U test was used to compare the effectiveness and safety of the treated facial sides at each session. A *p*‐value of < 0.05 was considered statistical significance.

## 3 Results

### 3.1 Demographic and Clinical Characteristics

The mean age of participants was 50.3 years (SD = 8.0), and they were all female. Fitzpatrick skin phototypes were 20% type III (*n* = 6) and 80% type IV (*n* = 24). Skin types were primarily dry (53.33%, *n* = 16), followed by usual (20%, *n* = 6), mixed (16.67%, *n* = 5), and oily (10%, *n* = 3). All had melasma for over a year. Previous treatments included laser therapy for 50% (*n* = 15), Cysteamine cream for 20% (*n* = 6), Kligman formula, and tranexamic acid for 3.33% each (*n* = 1), and 40% (*n* = 12) had not received any listed treatments (Table 1).

**TABLE 1:** Clinical characteristics and demographics of the study population.

Characteristics	Participants (*n* = 30)	
Age, mean ± SD	50.3 ± 8.0	
Gender (male/female)	0/30	
Fitzpatrick skin phototype (III/IV)	6/24	
Skin type		
Dry	53.3%	(16/30)
Mixed	16.7%	(5/30)
Oil	10.0%	(3/30)
Normal	20.0%	(6/30)
Duration > 1 year	100.0%	(30/30)
Previous treatment		
Laser	50.0%	(15/30)
Cysteamine cream	20.0%	(6/30)
Kligman's formula	3.3%	(1/30)
Tranexamic acid	3.3%	(1/30)
Underlying disease		
Vitiligo	26.7%	(8/30)
Hypertension	6.7%	(2/30)
Hyperlipidemia	6.7%	(2/30)
Hypothyroidism	3.3%	(1/30)

### 3.2 Evaluation of Treatment Protocols

In the study, participants received RFM treatment for the whole face coupled with an in‐office cysteamine split face study and randomization of the home‐based topical cysteamine cream or placebo vehicle control. The group categorization is as follows:

**Table:** 

	Cysteamine	
Group A	In‐office serum	Home‐based Intensive Cream
Group B	—	Home‐based Intensive Cream
Group C	In‐office serum	—
Group D	—	—

*Note:* Remarks:—indicating placebo vehicle control.

### 3.3 Hemi‐mMASI Score

**FIGURE 1:**
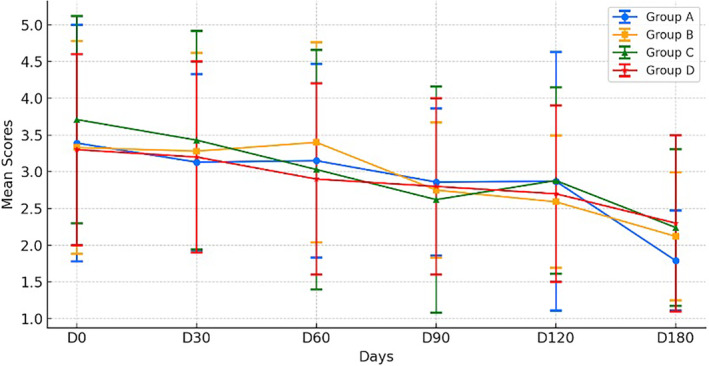
Trend of mean Hemi‐mMASI values across four groups throughout the trials. Group A combined in‐office and home topical cysteamine treatments. Group B used only intensive home treatment. Group C received only in‐office cysteamine treatment. Group D served as a control, receiving RFM.

Figure 1 shows the changes in Hemi m‐MASI scores for each treatment group over six sessions. At 180 days after treatment follow‐up, Group A's scores decreased from a mean of 3.39 ± 1.61 to 1.79 ± 0.68, indicating improvements in melasma. Group B's scores also reduced, from 3.33 ± 1.45 to 2.12 ± 0.87. Group C, starting at 3.71 ± 1.41, had fluctuating scores that eventually dropped to 2.24 ± 1.07. Group D's scores went from 3.28 ± 1.29 to 2.33 ± 1.19, with the slightest improvement.

Group A's improvements were statistically significant by session six (*t* = 3.662, *p* = 0.003), as were Group B's (*t* = 3.2, *p* = 0.008), highlighting the effectiveness of ongoing topical cysteamine use. Group C showed significant improvements from session three (*t* = 3.005, *p* = 0.009) onwards, while Group D's improvements were significant only by session six (*t* = 2.4, *p* = 0.034).

### 3.4 VISIA Score Assessments

**FIGURE 2:**
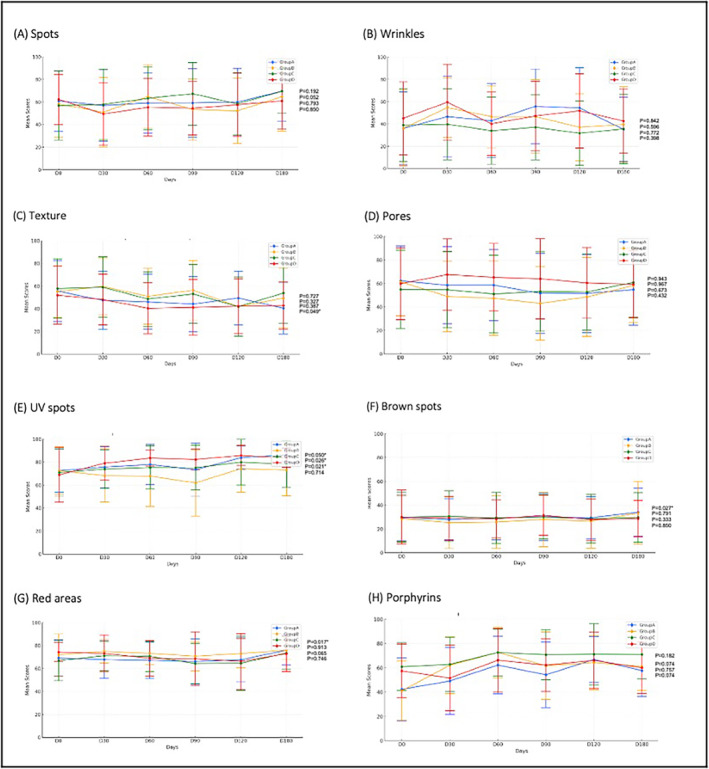
VISIA score trends across four groups during the trial period.

Figure 2 illustrates the skin parameter changes measured by VISIA imaging analysis in Group A. In Group A, there was a significant wrinkle reduction at the fourth (*t* = −2.389, *p* = 0.033) and fifth (*t* = −2.357, *p* = 0.035) sessions, and texture improvements at the fourth (*t* = 2.389, *p* = 0.033), fifth (*t* = 2.157, *p* = 0.05), and sixth (*t* = 2.19, *p* = 0.049) sessions. UV spots decreased significantly by the fifth (*t* = −2.739, *p* = 0.017) and sixth (*t* = −2.176, *p* = 0.05) sessions, but red areas increased at the sixth session (*t* = 2.772, *p* = 0.017).

Group B had skin texture improvements by the fifth session (*t* = 2.237, *p* = 0.045) and porphyrin reduction by the third session (*t* = −3.171, *p* = 0.007). Group C had UV spot reductions at the third (*t* = −2.384, *p* = 0.032), fifth (*t* = −3.396, *p* = 0.005), and sixth (*t* = −2.65, *p* = 0.021) sessions. Group D demonstrated UV spot reduction at the third (*t* = −2.374, *p* = 0.032), fourth (*t* = −2.334, *p* = 0.036), fifth (*t* = −2.957, *p* = 0.01), and sixth (*t* = −2.496, *p* = 0.026) sessions.

### 3.5 Safety Profiles

Erythema was the most prevalent side effect in 26.7% of Groups A (4/15) and 20.0% in Groups B, C, and D (3/15 each). Scaling appeared in 6.7% of Groups A and B (1/15 each) but not in Groups C or D. Allergic reactions were exclusive to Group A (6.7%, 1/15). Pain was reported in Group B (13.3%, 2/15) and Group D (6.7%, 1/15) but not in Groups A or C. Dryness was noted only in Group B (6.7%, 1/15). Acne was reported only in Group C (6.7%, 1/15), and itching was specific to Group D (6.7%, 1/15). (Table 2).

**TABLE 2:** Safety profile.

	Group A (*n* = 15)		Group B (*n* = 15)		Group C (*n* = 15)		Group D (*n* = 15)	
Erythema	26.7%	(4/15)	20.0%	(3/15)	20.0%	(3/15)	20.0%	(3/15)
Scaling	6.7%	(1/15)	6.7%	(1/15)	0.0%	(0/15)	0.0%	(0/15)
Allergic	6.7%	(1/15)	0.0%	(0/15)	0.0%	(0/15)	0.0%	(0/15)
Pain	0.0%	(0/15)	13.3%	(2/15)	0.0%	(0/15)	6.7%	(1/15)
Dryness	0.0%	(0/15)	6.7%	(1/15)	0.0%	(0/15)	0.0%	(0/15)
Acne	0.0%	(0/15)	0.0%	(0/15)	6.7%	(1/15)	0.0%	(0/15)
Itch	0.0%	(0/15)	0.0%	(0/15)	0.0%	(0/15)	6.7%	(1/15)

## 4 Discussion

The findings from this study significantly enhance the current understanding of melasma treatments, emphasizing the effects of combining RFM with topical cysteamine. The observed differences in treatment outcomes highlight the effectiveness of these approaches, both separately and in combination, providing valuable insights for clinical application.

**FIGURE 3:**
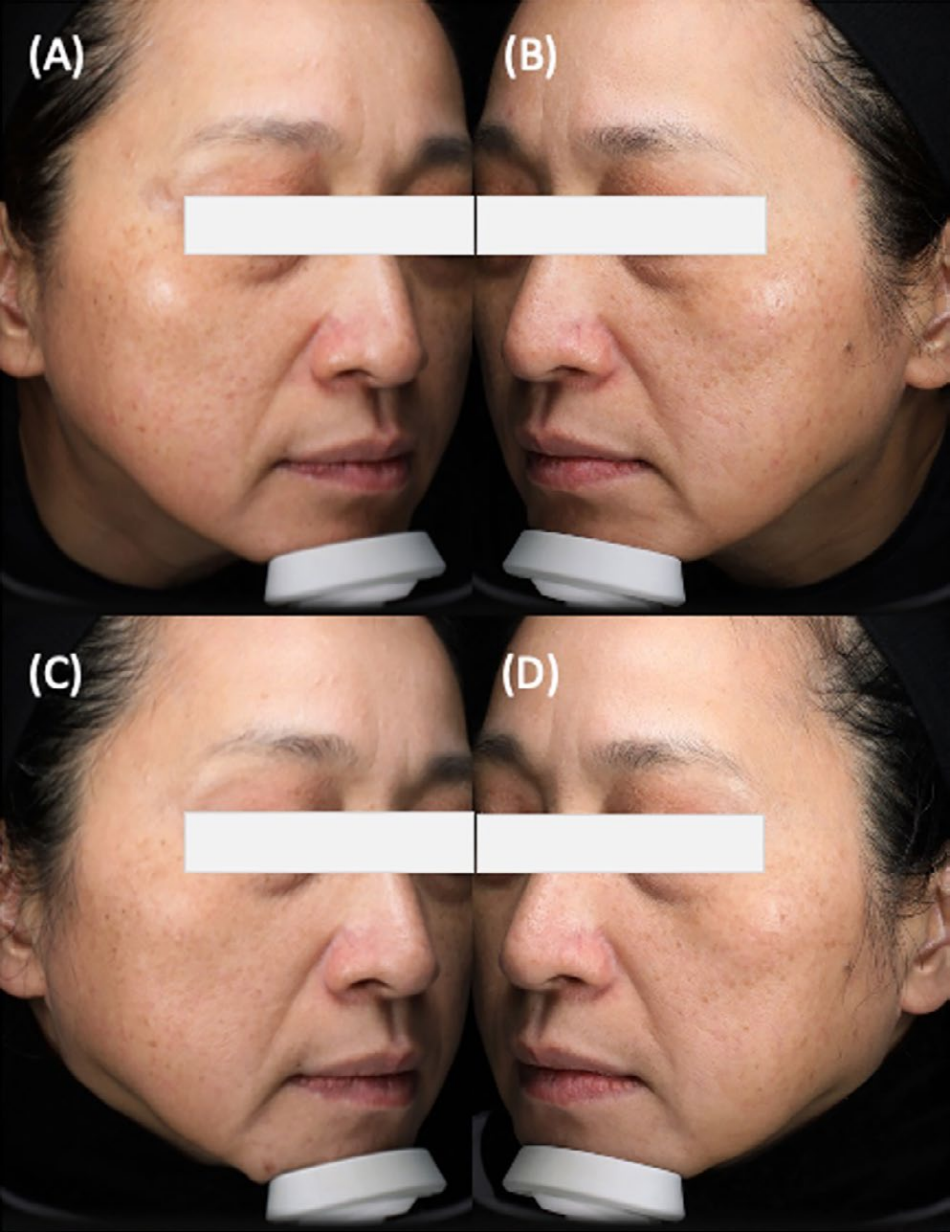
Treatment progression and outcomes for melasma. (A) and (C) show the right side of the face, which received RFM and both in‐office Cysteamine serum and home‐based Cysteamine cream application. (B) and (D) depict the left side, treated solely with RFM and home‐based Cysteamine cream. Panels (A) and (B) capture the baseline condition before treatment. In contrast, panels (C) and (D) illustrate the results at the 180‐day follow‐up, demonstrating the differential effects of adding in‐office treatment to home‐based therapy versus exclusive home‐based therapy on melasma.

**FIGURE 4:**
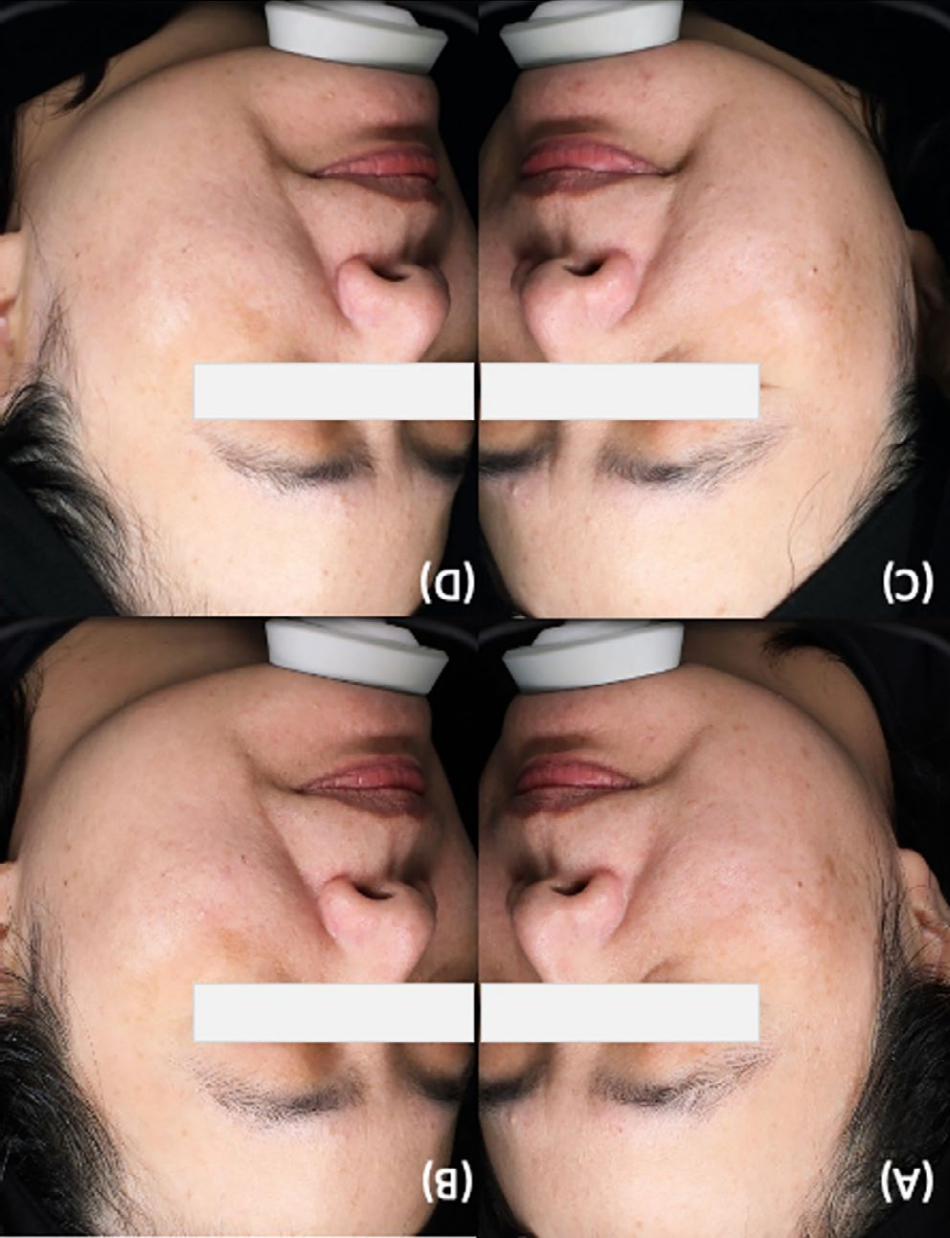
Treatment progression and outcomes for melasma. (A) and (C) depict the right side of the face, which was treated with in‐office RFM and Cysteamine serum. (B) and (D) show the left side of the face, which was treated with RFM. Panels (A) and (B) provide the baseline state before any intervention. In contrast, Panels (C) and (D) represent the outcomes at the 180‐day follow‐up, highlighting the effects of RFM and in‐office Cysteamine serum treatment compared to RFM alone.

Hemi m‐MASI score trends revealed improvements in melasma severity for all groups, with Group A exhibiting the most significant reduction. (Figures 3 and 4) The downward trajectory of Hemi m‐MASI scores suggests the treatments were effective, particularly for Group A, which underwent both in‐office and home‐based cysteamine treatments. Groups B and C also saw improvements, albeit less markedly. Group D's minimal score reduction confirms the supplemental role of cysteamine in enhancing melasma treatment outcomes. The early and sustained improvements in Group C underscore the effectiveness of in‐office cysteamine application in combination with RFM. The results indicate that the application method and patient compliance are crucial to successful outcomes, underlining the necessity for thorough patient education regarding correct application methods for home treatments. Incorrect application of cysteamine by patients may potentially diminish its efficacy.

The results of our study support the hypothesis that combining RFM with cysteamine enhances therapeutic outcomes by leveraging the complementary mechanisms of each modality. RFM increases the dermal absorption of cysteamine, thereby amplifying its ability to reduce melanin synthesis via tyrosinase inhibition [12, 17]. Additionally, RFM‐induced neocollagenesis helps repair the dermal damage associated with melasma, providing a more holistic approach to treatment. This synergistic effect could explain the significant improvement in Group A, where both in‐office RFM and home‐based cysteamine were used. These findings align with a prior study, which demonstrated that RFM could yield improvements in melasma at a 6‐month assessment point [11]. However, the study above documented melasma recurrence in three patients at 2, 3, and 4 months post‐treatment [11]. Our results further indicated that the adjunctive use of topical cysteamine may amplify the efficacy of RFM in treating melasma. This combination approach suggests a synergistic effect to address the overproduction of melanin and promote dermal remodeling and improved skin texture [9].

Interestingly, the VISIA analysis yielded results that diverge slightly from those measured by the mMASI score, highlighting the comprehensive benefits of RFM combined with topical cysteamine. Specifically, Group A demonstrated significant reductions in UV spots alongside notable improvements in skin wrinkles and texture, suggesting an enhanced capacity for aesthetic enhancement and mitigating UV radiation‐induced skin damage. Conversely, Group B exhibited improvements in skin texture and porphyrin levels at specific intervals, lacking consistency. This variability may suggest potential issues with the home‐based application of cysteamine, such as incorrect application techniques or timing, which could compromise the efficacy of the treatment or potentially diminish the benefits of RFM. In contrast, Group C recorded improvements in UV spots, pointing to the advantages of professional, in‐office application of cysteamine, possibly due to more precise application or improved absorption post‐RFM.

The observed incremental efficacy of Group D, treated exclusively with RFM, augments the existing literature endorsing RF's utility in managing melasma [17]. The VISIA score analysis for Group D, mirroring the UV spot reduction achievements of Group C, delineates RF's autonomous effectiveness in mitigating UV‐induced skin alterations. The observed improvements extending beyond melasma may be attributed to the dermal rejuvenation effects of RFM. The application of radiofrequency energy is known to encourage neocollagenesis and contribute to restoring the basement membrane in skin affected by melasma [18]. This suggests that RFM addresses pigmentation issues and enhances the skin's overall structural integrity and appearance [19, 20].

In our study, no severe side effects were reported, a finding that is consistent with previous research indicating that RFM and cysteamine treatments are safe [12, 17]. The higher erythema rate in Group A could be associated with the more aggressive treatment protocol of in‐office plus intensive home‐based cysteamine. The reports of pain in Group B suggest a possible irritant effect of intensive cysteamine application when used as a sole post‐procedure treatment at home. In contrast, the absence of pain in Group C indicates that in‐office application of cysteamine might be better tolerated. These results demonstrate the need for a careful balance between maximizing treatment efficacy and minimizing adverse effects.

The limitations of this study encompass a restricted number of participants, challenges in monitoring patient compliance with at‐home treatment regimens, and the impact of seasonal transitions from spring to summer, which entails increased UV exposure. Additionally, there was a lack of quantifiable sunscreen monitoring among participants. These factors collectively contribute to potential variability in treatment outcomes and underscore the need for meticulous consideration of external variables that may influence the efficacy of melasma treatments. Despite significant improvements observed in MASI score and VISIA skin imaging analysis, patient reported improvements remained non‐significant across all four treatment groups. This suggests that melasma patients may have higher expectations for clinical results.

Our research substantiates the effectiveness of combining RFM and topical cysteamine therapies for treating melasma, especially involving professional, in‐office applications. It highlights the potential of this synergistic approach to enhance treatment outcomes and emphasizes the necessity for additional investigations into optimizing treatment protocols and improving patient adherence to these therapies.

## Author Contributions

Y.‐W.T., J.‐H.L., and C.Y.N. performed the research. C.Y.N. designed the research study. C.‐H.L. and C.Y.N. contributed essential reagents or tools. Y.‐J.L. and T.‐L.L. analyzed the data. Y.‐W.T. and T.‐L.L. wrote the paper.

## Consent

The IRB approves the waiver of the participants’ consent.

## Conflicts of Interest

C.Y.N. received an honorarium for lecturing for Scientis Pharma. The other authors declare that the research was conducted without any commercial or financial relationships that could be construed as a potential conflict of interest.

## IRB Approval Status

Reviewed and approved by the Chang Gung Medical Foundation Institutional Review Board; approval 2204270018.

## Supporting information


Figure S1.

## Data Availability

The authors have nothing to report.
