# Supplementary figures and images for: Efficacy and Safety of Combination Therapy of Microneedling Radiofrequency, In‐Office and Home‐Based Topical Cysteamine in Refractory Melasma: A Split Face, Vehicle‐Control, Randomized Control Trial
Source: J Cosmet Dermatol. 2024 Nov 22;24(2):e16661. doi: 10.1111/jocd.16661 (PMC11845957; doi:10.1111/jocd.16661)

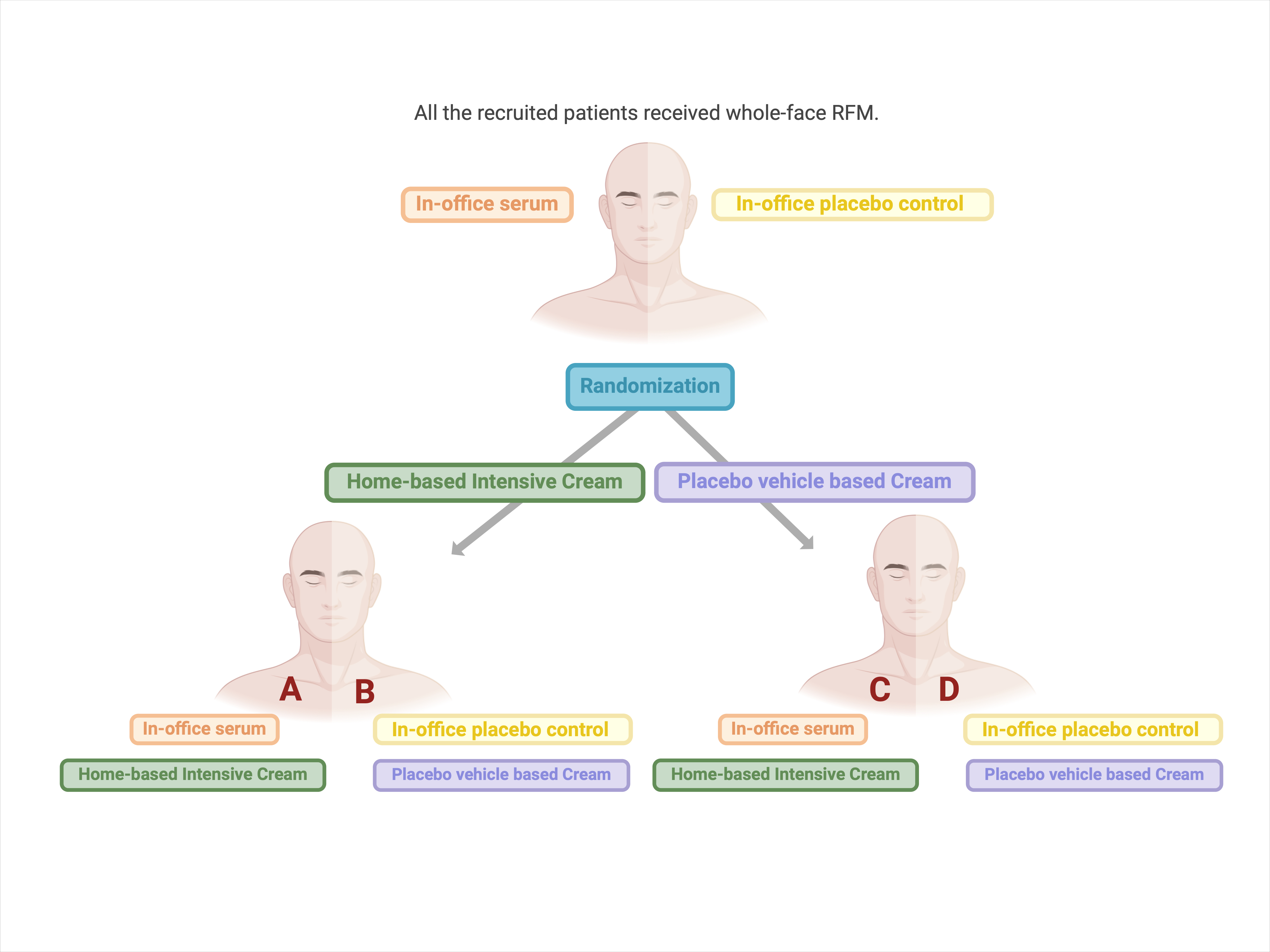

Supplement: Supplementary file 1 — Figure S1. [file JOCD-24-e16661-s001.jpeg]
